# Whole exome sequencing coupled with unbiased functional analysis reveals new Hirschsprung disease genes

**DOI:** 10.1186/s13059-017-1174-6

**Published:** 2017-03-08

**Authors:** Hongsheng Gui, Duco Schriemer, William W. Cheng, Rajendra K. Chauhan, Guillermo Antiňolo, Courtney Berrios, Marta Bleda, Alice S. Brooks, Rutger W. W. Brouwer, Alan J. Burns, Stacey S. Cherny, Joaquin Dopazo, Bart J. L. Eggen, Paola Griseri, Binta Jalloh, Thuy-Linh Le, Vincent C. H. Lui, Berta Luzón-Toro, Ivana Matera, Elly S. W. Ngan, Anna Pelet, Macarena Ruiz-Ferrer, Pak C. Sham, Iain T. Shepherd, Man-Ting So, Yunia Sribudiani, Clara S. M. Tang, Mirjam C. G. N. van den Hout, Herma C. van der Linde, Tjakko J. van Ham, Wilfred F. J. van IJcken, Joke B. G. M. Verheij, Jeanne Amiel, Salud Borrego, Isabella Ceccherini, Aravinda Chakravarti, Stanislas Lyonnet, Paul K. H. Tam, Maria-Mercè Garcia-Barceló, Robert M. W. Hofstra

**Affiliations:** 1 Department of Surgery, Li Ka Shing Faculty of Medicine, The University of Hong Kong, Hong Kong, SAR China; 2 Centre for Genomic Sciences, Li Ka Shing Faculty of Medicine, The University of Hong Kong, Hong Kong, SAR China; 3 Department of Neuroscience, section Medical Physiology, University of Groningen, University Medical Center Groningen, Groningen, The Netherlands; 4 Department of Clinical Genetics, Erasmus University Medical Center, PO Box 2040, 3000CA Rotterdam, The Netherlands; 5 Department of Genetics, Reproduction and Fetal Medicine, Institute of Biomedicine of Seville (IBIS), University Hospital Virgen del Rocío/CSIC/University of Seville, Seville, Spain; 6 Centre for Biomedical Network Research on Rare Diseases (CIBERER), Seville, Spain; 7 McKusick-Nathans Institute of Genetic Medicine, Johns Hopkins University School of Medicine, Baltimore, USA; 8 Department of Medicine, School of Clinical Medicine, University of Cambridge, Addenbrooke’s Hospital, Cambridge, UK; 9 Erasmus Center for Biomics, Erasmus Medical Center, Rotterdam, The Netherlands; 10 Stem Cells and Regenerative Medicine, Birth Defects Research Centre, UCL Institute of Child Health, London, UK; 11 UOC Genetica Medica, Istituto Gaslini, Genoa, Italy; 12 Department of Biology, Emory University, Atlanta, USA; 13 Laboratory of embryology and genetics of human malformations, INSERM UMR 1163, Institut Imagine, Paris, France; 14 Department of Genetics, Paris Descartes-Sorbonne Paris Cité University, Hôpital Necker-Enfants Malades (APHP), Paris, France; 15 Department of Biochemistry and Molecular Biology, Faculty of Medicine, Universitas Padjadjaran, Bandung, Indonesia; 16 Department of Genetics, University of Groningen, University Medical Center Groningen, Groningen, The Netherlands

**Keywords:** De novo mutations, Hirschsprung disease, Neural crest, ENS

## Abstract

**Background:**

Hirschsprung disease (HSCR), which is congenital obstruction of the bowel, results from a failure of enteric nervous system (ENS) progenitors to migrate, proliferate, differentiate, or survive within the distal intestine. Previous studies that have searched for genes underlying HSCR have focused on ENS-related pathways and genes not fitting the current knowledge have thus often been ignored. We identify and validate novel HSCR genes using whole exome sequencing (WES), burden tests, in silico prediction, unbiased in vivo analyses of the mutated genes in zebrafish, and expression analyses in zebrafish, mouse, and human.

**Results:**

We performed de novo mutation (DNM) screening on 24 HSCR trios. We identify 28 DNMs in 21 different genes. Eight of the DNMs we identified occur in RET, the main HSCR gene, and the remaining 20 DNMs reside in genes not reported in the ENS. Knockdown of all 12 genes with missense or loss-of-function DNMs showed that the orthologs of four genes (DENND3, NCLN, NUP98, and TBATA) are indispensable for ENS development in zebrafish, and these results were confirmed by CRISPR knockout. These genes are also expressed in human and mouse gut and/or ENS progenitors. Importantly, the encoded proteins are linked to neuronal processes shared by the central nervous system and the ENS.

**Conclusions:**

Our data open new fields of investigation into HSCR pathology and provide novel insights into the development of the ENS. Moreover, the study demonstrates that functional analyses of genes carrying DNMs are warranted to delineate the full genetic architecture of rare complex diseases.

**Electronic supplementary material:**

The online version of this article (doi:10.1186/s13059-017-1174-6) contains supplementary material, which is available to authorized users.

## Background

Hirschsprung disease (HSCR) is the most common form of congenital obstruction of the bowel, with an incidence of ~1/5000 live births. The incidence varies significantly between ethnic groups, the highest being in Asia (2.8/10,000 live births) [1]. HSCR results from a failure of the neural crest (NC) cells, which give rise to the enteric nervous system (ENS), to migrate, proliferate, differentiate, or survive in the bowel wall, resulting in aganglionosis of the distal part of the gastrointestinal tract. This results in clinically severe and sometimes life-threatening bowel obstruction. As HSCR is a highly heritable disorder, genetic variation (mutations) in the genomes of these patients must largely explain disease development. So far more than 15 HSCR susceptibility genes, six linkage regions [1], and three associated loci [2, 3] have been found. The genes identified belong to a limited number of pathways relevant to the development of the ENS, among which the RET and the endothelin pathways are the most important. *RET* (encoding a tyrosine kinase) is the major gene with >80% of all known mutations. These have been mainly identified in ~50% of familial (mostly long-segment HSCR (L-HSCR), total colonic aganglionsis (TCA)) and up to 20% of sporadic (mostly short-segment HSCR (S-HSCR)) cases [4]. However, the identified genes and variants explain no more than 25% of the overall genetic risk of all HSCR cases [2, 3]. These findings indicate that the majority of the disease risk must be due to as yet unidentified rare or common variants in the known HSCR genes or, more likely, variants in yet unknown genes, acting alone or in combination.

The most popular approach to the analysis of whole exome sequencing (WES) data includes selecting genes that can be functionally linked to the pathways already known to be involved in the disease under study. Variants in genes totally unlinked to the known genes or pathways are largely neglected. This study aimed to determine the contribution of rare exonic, non-synonymous DNMs to HSCR without any a priori selection. Therefore, not only did we perform “standard” exome sequencing analyses, followed by burden tests and in silico prediction, but we also carried out an unbiased in vivo analysis of the mutated genes in a zebrafish model.

## Results

### Identification of de novo mutations

In total 24 HSCR trios (Additional file 1: Table S1) were included for WES analyses (Additional file 2: Figures S1 and S2). Sequencing metrics after the standard analytical pipeline (Additional file 2: Figure S2) are detailed in Additional file 3: Table S2 and Additional file 2: Figure S3. Specifically, the coverage of the targeted sequences per sample ranged from 18× to 74× (average 46×), and the targeted exome was covered by at least ten sequence reads which ranged from 65 to 98% (average 88%). All these quality metrics or statistics showed data quality at exonic regions that were comparatively good for trios from different platforms or resources and justified our unbiased searching of de novo mutations in the following stages.

After validation, a total of 28 DNMs in 14 patients were identified (Table 1; Additional file 1: Table S1; Additional file 4: Table S3). The overall DNM rate per individual was 1.2 per exome per generation (Poisson distribution with *λ* = 1.2; Kolmogorov–Smirnov test, *p* = 0.893; Additional file 2: Figure S4), which is in accordance with the expected rate in the general population [5]. Several studies found that patients have a significantly higher fraction of loss of function (LOF) DNMs than healthy controls [6, 7]. In our HSCR patient cohort, the LOF DNM rate (8 out of 24 trios) is significantly higher than that of healthy trios (4 out of 54 trios; binomial test *p* = 0.011) or unaffected siblings of neuropsychiatric patients (54 out of 677 trios; binomial test *p* = 0.001) (Additional file 5: Table S4) [6, 8–11]; however, the enrichment of non-*RET* LOF DNMs (3 out of 24 trios) in our trios is not significant. These 28 DNMs were distributed among 21 genes. Eight DNMs were in *RET*, the major HSCR gene [12]. The observed *RET* DNM rate (0.33/trio) was significantly higher (*p* < 2 × 10^−16^) than that modeled in the general population (0.000133/trio) according to Samocha et al. [13].

**Table 1:** De novo mutations in Hirschsprung disease probands

Trio	Phenotype	Gene	De novo mutation	Type	MAF (dbSNP137/ESP6500/ExAC)^a^
1	L, F	***RET***	3splicing9 + 1	Splicing	N/N/N
		*RBM25*	c.474C > T:p.L158L	Synonymous	N/N/N
2	L, F	***RET***	c.2511_2519delCCCTGGACC:p.S837fs	Frameshift	N/N/N
		*COL6A3*	c.3327C > T:p.H1109H	Synonymous	4.2E-4 (rs114845780)/N/1.2E-4
3	L, F	***RET***	c.1818_1819insGGCAC:p.Y606fs	Frameshift	N/N/N
4	L, F	*DAB2IP*	c.2339C > T:p.T780M^b^	Missense	N/N/2.8E-3
		*ISG20L2*	c.961G > A:p.G321R	Missense	N/N/N
		*MED26*	c.675C > T:p.A225A	Synonymous	N/N/N
		*NCLN*	c.496C > T:p.Q166X^b^	Nonsense	N/N/N
		*NUP98*	c.5207A > G:p.N1736S	Missense	N/N/N
		*VEZF1*	c.584C > T:p.S195F	Missense	N/N/N
		*ZNF57*	c.570C > T:p.D190D	Synonymous	N/N/N
5	L, F	***RET***	c.1761delG :p.G588fs	Frameshift	N/N/N
		*SCUBE3*	c.1493A > T:p.N498I	Missense	N/N/N
6	L, M	*AFF3*	c.1975G > C:p.V659L	Missense	N/N/N
		*PLEKHG5*	c.2628G > T:p.T876T	Synonymous	N/N/9.1E-6
7	L, M	*KDM4A*	c.26A > G:p.N9S	Missense	N/N/N
8	L, M	*MAP4*	c.3351C > T:p.G1117G	Synonymous	N/N/9.2E-6
9	L, F	***RET***	c.1858 T > C:p.C620R	Missense	0 (rs77316810)/N/N
10	TCA, M	*CKAP2L*	c.555_556delAA:p.E186fs	Frameshift	N/2E-5/2.5E-5
11	L, F	***RET***	c.409 T > G:p.C137G	Missense	N/N/N
		*HMCN1*	c.10366G > A:p.A3456T	Missense	N/N/N
		*TUBG1*	c.699 T > C:p.S233S	Synonymous	N/N/8.2E-6
12	L, F	*CCR2*	c.848 T > A:p.L283Q	Missense	N/N/N
		*DENND3*	c.1921delT:p.K640fs	Frameshift	N/N/N
13	L, F	***RET***	c.1710C > A:p.C570X	Nonsense	N/N/N
14	L, F	***RET***	c.526_528delGCA:p.R175del	Non-frameshift	N/N/N
		*TBATA*	c.157C > T:p.R53C	Missense	N/N/4.1E-5

Genes in bold indicate patients carrying de novo *RET* mutations. Underlines genes are genes giving a HSCR-like phenotype in zebrafish.

*F* female, *L* long-segment HSCR, *M* male, *TCA* total colonic aganglionsis

^a^Minor allele frequency (MAF) in dbSNP137, ESP database or ExAC database, with “N” standing for no data available.

^b^Mosaic mutation

One patient carried seven DNMs, two of which (*NCLN* and *DAB2IP*) were mosaic (Additional file 2: Figure S5). This is in line with a recent report stating that 6.5% of all DNMs are mosaic and occur post-zygotically [14]. Within the 24 patients, we looked for inherited rare damaging variants in the 21 genes carrying DNMs. Inherited damaging mutations were found in *RET*, *HMCN1*, *PLEKHG5*, *MAP4*, *SCUBE3*, and *KDM4A* (Additional file 6: Table S5). Neither de novo nor inherited copy number variants (CNVs) were detected.

### Determining pathogenicity of the DNMs in silico

We mainly followed the guidelines from Veltman and Brunner [15] and MacArthur et al. [16] to determine the pathogenicity of the variants and genes found in this study. Firstly, to establish whether DNM genes carried significantly more rare variants in HSCR patients than in controls, we used WES data from the 20 eligible HSCR trio-probands, 28 additional HSCR patients, and 212 control individuals to calculate the variation burden per gene. Nine of the 21 genes (*RET*, *KDM4A*, *HMCN1*, *MAP4*, *NUP98*, *AFF3*, *COL6A3*, *CCR2*, and *CKAP2L*) were mutated in different sites in different HSCR patients (Additional file 7: Table S6). Meta-analysis of our gene burden tests showed that *RET* and *CKAP2L* were enriched for rare damaging variants in HSCR (uncorrected *p* < 0.05; Table 2; Additional file 7: Table S6), though our sample size is underpowered for genome-wide statistical tests (Additional file 2: Figure S6). However, crosschecking these 21 genes in another in-parallel HSCR WES (190 cases, 740 controls) revealed that only *RET* was significantly overrepresented with deleterious variants (*p* < 0.001; A. Chakravarti, manuscript in preparation).

**Table 2:** Genes carrying de novo mutations

Gene	Number of amino acids	Co-occurrence with *RET* DNM	Burden test meta-analyses (uncorrected *p* value)	Gene constraint prediction (AGTU; ExAC)^a^	Zebrafish ENS phenotype	Gut expression (human; mouse; zebrafish)^b^
*PLEKHG5*	1062	No	0.3997	No; No	NT	Yes; Yes; ND
*KDM4A*	1064	No	0.1190	No; Yes	No	Yes; Yes; ND
*ISG20L2*	353	No	0.4949	No; No	No	Yes; Yes; ND
*HMCN1*	5635	Yes	0.9789	No; No	No	Yes; Yes; ND
*AFF3*	1226	No	0.4745	No; Yes	No	Yes; Yes; ND
*CKAP2L*	745	No	0.0178	No; No	No	Yes; Yes: ND
*COL6A3*	3177	Yes	0.6398	No; No	NT	Yes; Yes; ND
*CCR2*	374	No	0.4745	No; No	No	Yes; Yes; ND
*MAP4*	1152	No	0.4851	No; Yes	NT	Yes; No; ND
*SCUBE3*	993	Yes	0.7133	Yes; Yes	No	Yes; Yes; ND
*DENND3*	1198	No	0.5977	No; No	Yes	Yes; Yes; Yes
*DAB2IP*	1189	No	0.9819	No; Yes	No	Yes; Yes; ND
*RET*	1114	-	0.0078	No; Yes	Yes	Yes; Yes; ND
*TBATA*	351	Yes	0.8028	NA; No	Yes	No; Yes; Yes
*NUP98*	1817	No	0.7243	No; Yes	Yes	Yes; Yes; Yes
*RBM25*	843	Yes	0.0846	Yes; Yes	NT	Yes; Yes; ND
*TUBG1*	451	Yes	1.0000	Yes; Yes	NT	Yes; Yes; ND
*VEZF1*	521	No	0.6717	No; Yes	No	Yes; Yes; ND
*ZNF57*	555	No	0.3808	No; No	NT	Yes; No; ND
*NCLN*	563	No	0.4949	No; No	Yes	Yes; Yes; Yes
*MED26*	600	No	1.0000	No; Yes	NT	Yes; Yes; ND

^a^Genes evolutionarily constrained as per AGTU’s server and ExAC database: “No” for not constrained, “Yes” for constrained, “NA” for not available.

^b^Data from in-house human induced pluripotent stem cell-derived neural crest, mouse expression data, and RT-PCR and in situ hybridization in zebrafish; *ND* not done.

*NT* not tested (gene carries synonymous mutation and/or has no ortholog in zebrafish)

Besides the eight LOF mutations, six out of twelve missense mutations were consistently predicted to be deleterious (Additional file 8: Table S7). As for the seven synonymous DNMs, we found no in silico evidence indicating that those changes interfered with splicing and/or RNA structure (Additional file 8: Table S7). After checking those genes with DNMs against the ATGU’s gene look-up server [13] and the Exome Aggregation Consortium (ExAC) Browser [17], in total 11 genes (Table 2) were identified as evolutionarily constrained where variants are more likely to be deleterious [13]. Next we checked whether the genes with DNMs were functionally related to each other and/or to the signaling networks known to govern ENS development. Although no direct in silico interactions were found among the 21 genes, *ISG20L2* and *MAP4* showed more indirect interactions with other genes with DNMs than that expected by chance (*p* = 0.0063 and *p =* 0.0167, respectively). A list of 116 ENS-related genes (Additional file 9: Table S8) was used to study the functional link between DNM genes (other than *RET*) and ENS. Only a single interaction was identified (*COL6A3* interacts with *ITGB1*). Ingenuity Pathway Analysis identified additional direct and indirect relationships with ENS-related genes for *MAP4*, *COL6A3*, *RBM25*, and *TUBG1* (Additional file 2: Figure S7). All genes carrying DNMs were either expressed in human induced pluripotent stem cell (iPSC)-derived enteric neuron precursors or in primary murine enteric neuron precursors (Table 2).

### Determining pathogenicity of the DNMs in vivo

**Fig. 1:**
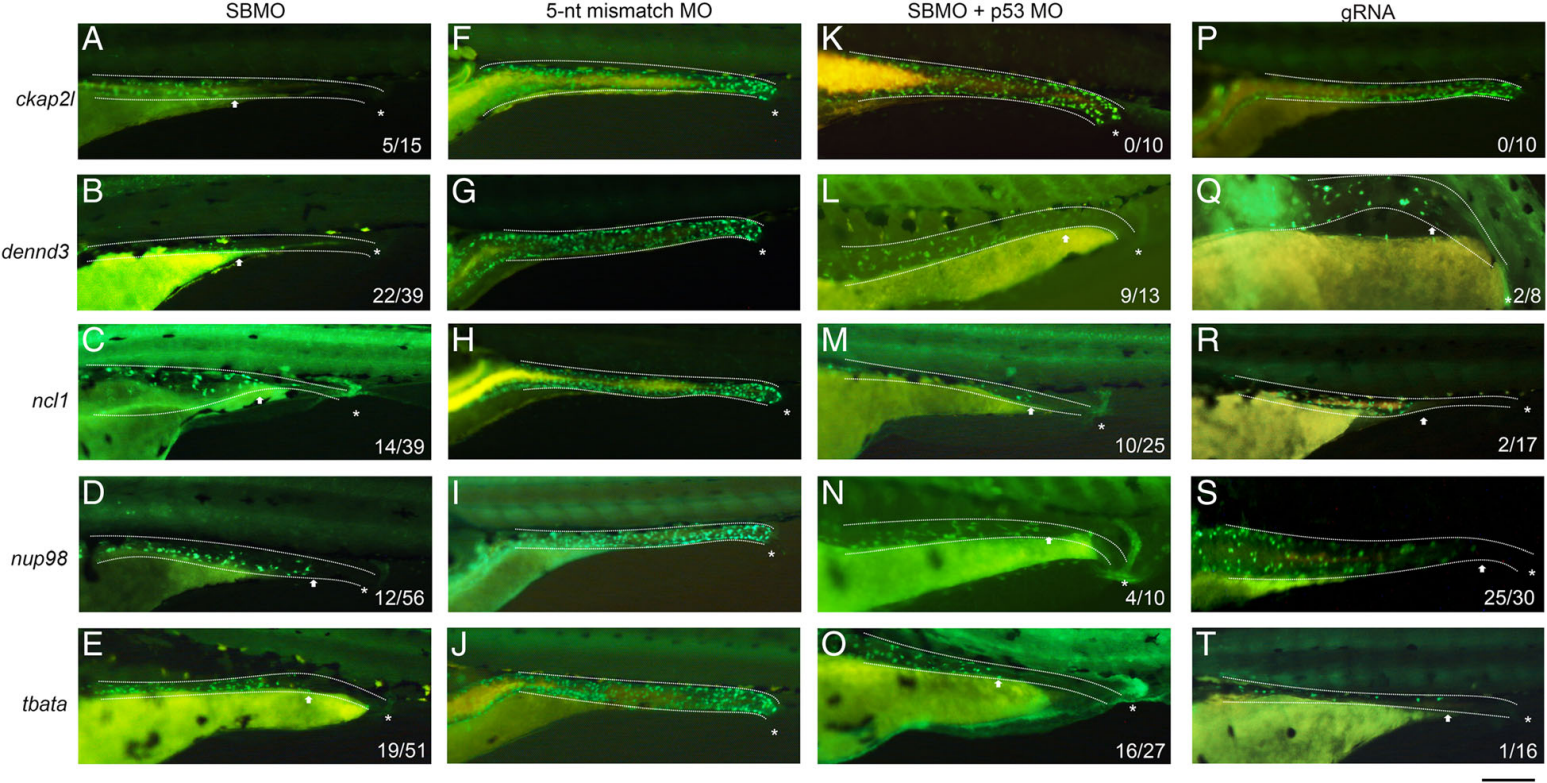
Pathogenicity analysis in vivo by morpholino gene knockdown and CRISPR/Cas9 knockout in zebrafish. Morpholino knockdown of *ckap2l*, *dennd3*, *ncl1*, *nup98*, and *tbata* resulted in a HSCR-like phenotype when compared to control (**a**–**j**). Kaede-expressing enteric neurons were absent in the distal intestine at 5 dpf. The number of embryos with phenotype out of the total number of embryos observed is shown. Co-injection of p53 morpholino reproduced the phenotype except *ckap2l*, indicating the loss of enteric neurons in *dennd3*, *ncl1*, *nup98*, and *tbata* knockdown was not the result of *p53*-induced apoptosis (**k**–**o**). The results were verified by CRISPR/Cas9 knockout of *ckap2l*, *dennd3a* and *b*, *ncl1*, *nup98*, and *tbata*, in which the HSCR-like phenotype was reproduced (**p**–**t**). *Dotted lines* outline the intestines. *Asterisks* indicate the position of the anus. *Arrows* indicate the position where the aganglionic region begins. *Scale bar* = 200 μm. MO morpholino, *nt* nucleotide

Thirteen genes had a LOF or missense mutation but were not obvious candidates for HSCR as there was no previous report linking these genes to ENS development or HSCR pathogenesis. We used the zebrafish model system to further investigate the function of these genes in ENS development. We used the model as previous studies have shown that morpholino-mediated knockdown of orthologs of known HSCR genes did result in an HSCR-like phenotype in zebrafish [3, 18]. Except *CCR2*, all 13 genes with nonsynonymous DNMs have zebrafish orthologs. Splice-blocking morpholinos (SBMOs) against the 12 genes were injected into *Tg(-8.3phox2b:Kaede)* transgenic zebrafish [19] embryos that express the fluorescent protein Kaede in enteric neuron precursors and differentiated enteric neurons, while its effect on gene transcription was confirmed by quantitative PCR (qPCR)/RT-PCR expression pattern. Initially, knockdown of five orthologs (*ckap2l*, *dennd3a* and *b*, *ncl1*, *nup98*, and *tbata*) resulted in a HSCR-like phenotype as enteric neurons were absent in the distal intestine of embryos 5 days post-fertilization (dpf) (Fig. 1a–e), while embryos injected with 5-nucleotide mismatch control morpholinos had normal ENS development with enteric neurons present along the entire length of the intestine (Fig. 1f–j). It was reported that morpholinos might induce target-independent apoptosis through p53 activation that leads to an unspecific off-target phenotype [20]. To confirm the HSCR-like phenotype observed in the morphants resulted from target-specific knockdown, we co-injected the SBMOs with p53 morpholino, which would inhibit p53 activity and thereby block the unspecific apoptosis. As a result, the phenotype could not be reproduced by *ckap2l* SBMO and p53 morpholino co-injection (Fig. 1k), suggesting the phenotype observed initially was an off-target effect. On the contrary, co-injection of p53 morpholino with *dennd3a* and *b*, *ncl1*, *nup98*, or *tbata* SBMOs resulted in the same phenotype (Fig. 1l–o), indicating the phenotype was not caused by unspecific apoptosis. qPCR analysis showed that the expression of *dennd3a*, *dennd3b*, *nup98*, and *tbata* was markedly reduced in the SBMO-injected embryos (Additional file 2: Figure S8). Intriguingly, there was no significant reduction in *ncl1* expression in the *ncl1* SBMO-injected embryos. Therefore, we further investigated it by performing RT-PCR on individual embryos and found that there was a large variation in *ncl1* expression between embryos injected with the SBMO, with some of them showing a clear reduction in *ncl1* transcript level (Additional file 2: Figure S9). Of the zebrafish orthologs that did not show a specific HSCR-like phenotype after SBMO injection, all demonstrated significant reductions in expression except for *aff3*, *scube3*, and *vezf1a* (Additional file 2: Figure S8). We verified the results by repeating the knockdown experiment with a second, non-overlapping translation-blocking morpholino (TBMO) against *dennd3a* and *b*, *ncl1*, *nup98*, or *tbata* and the HSCR-like phenotype was reproduced (data not shown). Overall, from the morpholino knockdown experiment we identified 4 out of 12 candidate genes that were important for ENS development and caused a HSCR-like phenotype when their functions were disrupted.

With the recent improvement in the CRISPR knockout protocol in zebrafish, which enabled phenotype analysis in guide-RNA (gRNA)-injected F0 larvae [21], we decided to carry out CRISPR knockout of *ckap2l*, *dennd3a* and *b*, *ncl1*, *nup98*, and *tbata* to further strengthen our data obtained from morpholino knockdown. We first tested the protocol by injecting *ret* gRNA and observed loss of enteric neuron phenotype in 5-dpf F0 larvae (data not shown). *ckap2l* gRNA did not cause a HSCR-like phenotype (Fig. 1p), reaffirming the interpretation that the initial observation in morpholino knockdown was an off-target effect. CRISPR knockout of *dennd3a* and *b*, *ncl1*, *nup98*, and *tbata* all resulted in the loss of enteric neurons in 5-dpf larvae (Fig. 1q–t). The presence of indel mutation at the target site was confirmed by T7E1 assay (Additional file 2: Figure S10). Therefore, we concluded that *DENND3*, *NCLN*, *NUP98*, and *TBATA* orthologs’ loss of function disrupted ENS development and caused a HSCR-like phenotype in vivo.

**Fig. 2:**
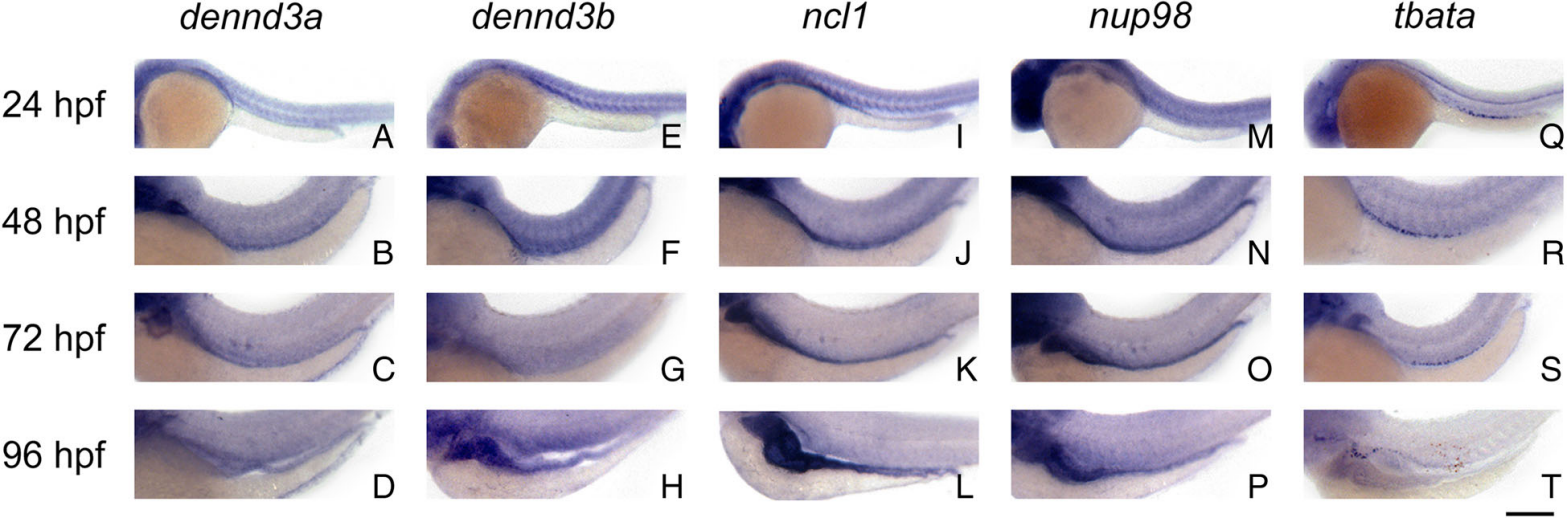
Temporal and spatial expression patterns of zebrafish orthologs. Whole mount in situ hybridized embryos hybridized with antisense riboprobes for *dennd3a* (**a**–**d**), *dennd3b* (**e**–**h**), *ncl1* (**i**–**l**), *nup98* (**m**–**p**), and *tbata* (**q**–**t**) at the indicated developmental stages. All columns show lateral views. Intestinal expression for all genes is apparent from 48 hpf onwards. *Scale bar* = 500 μm

Temporal analysis using RT-PCR revealed zebrafish orthologs (*dennd3a*, *dennd3b*, *ncl1*, and *nup98*) for *DENND3*, *NCLN*, and *NUP98* were maternally and zygotically expressed from 0–120 hpf while the *TBATA* ortholog (*tbata)* is only zygotically expressed from 24–120 hpf (Additional file 2: Figure S11). Whole mount in situ hybridization (WISH) analysis showed that the orthologs for all four genes were expressed in distinct spatial locations specifically in the intestine and the anterior central nervous system (CNS) from 24–96 hpf, suggesting a role not only in ENS development but also in the CNS (Fig. 2).

### Mutation profile of HSCR patients

Out of the 14 patients with DNM, eight carried mutations in RET and six in genes other than RET. Interestingly, one of the patients with RET DNM also harbored DNM in genes that recapitulate HSCR in zebrafish (TBATA), suggesting that, in humans, mutations in more than one gene might be necessary for the phenotype to develop. Among the six patients with no RET DNM, two (HSCR4 and HSCR12; Table 1) harbored functionally supported DNMs. Overall, we observed 33% (8 out of 24) diagnostic rate for *RET* mutations in all our patients, but 62.5% (5 out of 8) if considering only those non-prescreened trio probands. This is consistent with previous report on *RET* contribution to L-HSCR [4].

Besides, since both rare and common variants jointly contribute to HSCR, we examined the genetic profile (Additional file 6: Table S5) of our patients to assess the genetic background on which the DNMs reside. Each patient was investigated for the presence/absence of the common HSCR-associated *RET* allele (rs2435357T) [22] as well as for the presence of rare variants (inherited from unaffected parents) in a set of 116 ENS-related pre-selected genes (Additional file 4: Tables S3; Additional file 9: Table S8). We did observe common RET risk alleles and rare damaging mutations in ENS candidate genes in all patients (Additional file 6: Table S5), regardless of their DNM status. Whether common or rare RET risk alleles and/or rare mutations in ENS genes in the background contribute to the phenotype would need further research.

## Discussion

Over the past years a large number of papers have been published on de novo mutation screening in human diseases. This has resulted in the identification of many new disease-associated genes. Genes are considered as true disease causing when there is a significant excess of de novo mutations among unlinked patients. This works well for diseases that are relatively homogeneous or for which many patients can be investigated. For the more heterogeneous rare diseases for which only small cohorts are available this poses a problem. Often possible disease causing genes are found in a single patient. How to decide whether this finding is of importance? Expression of the gene in the relevant tissues can be considered as additional evidence, as is network analysis. However, making strong statements for private disease genes is, and will be, extremely difficult. It also results in a bias towards genes in the known disease-causing gene networks. Genes not fitting the current knowledge are often discarded as uninteresting. In the current study we wanted to take this all one step further.

Therefore, to assess if the mutated genes play a role in ENS development, we performed two rounds of functional analyses. We opted for an in vivo approach using the zebrafish model system. We knocked down the expression of zebrafish orthologs of 12 of the 13 genes in which loss-of-function or missense DNMs had been identified. Nine genes were successfully knocked down and four of them resulted in loss of neurons in the distal gut. It was noted that in some morphants the proportion of larvae displaying a HSCR-like phenotype was comparatively low. Although in most cases we could improve the efficacy by increasing the amount of morpholino injected, this also led to a higher death rate and more dysmorphic larvae caused by the general toxicity of the morpholino. Therefore, we chose the dose that caused the minimum death rate and dysmorphic rate with slightly lower, but still valid, efficacy. It should also be noted that in some of the morphants the gut development appeared to be affected. Perhaps it was not surprising as from the in situ hybridization results some of these genes seemed to be expressed in the surrounding intestinal tissues as well. Therefore, when hypothesizing the function of these genes in ENS development, in addition to the intrinsic effect on enteric neural crest cells, one should not rule out the possibility of the extrinsic influence via the intestinal tissues. Noteworthy, the SBMOs targeting three of the orthologs (aff3, scube3, and vezf1a) did not knockdown the target transcripts as expected, highlighting the limitation of morpholinos [23]. To strength our morpholino data we opted for CRISPR knockout, which allowed phenotype analysis in F0 larvae. The HSCR-like phenotype was reproduced in the CRISPR knockout of dennd3a and b, ncl1, nup98, and tbata. Although the presence of indel mutation was confirmed by T7E1 assay in all larvae injected with gRNA, the number of larvae displaying the phenotype was relatively low. We suspected this was mainly due to the varying CRISPR efficiency (number of indels present in the target gene over the total number of the target gene copy in a larva) between larvae.

For those four mutations from the newly validated genes (*DENND3*, *NCLN*, *NUP98*, and *TBATA*), two of them (*NCLN*:Q166* and *DENND3*:K640fs) are loss-of-function mutations that disrupt gene translation; the other two (NUP98: N1662S and TBATA:R53C) are predicted as highly deleterious by Polyphen2 and a logistic regression model that combine different predicted scores [24]. This in silico evidence (Table S8) strongly supports the potential impact of the mutations on their genes [15]; hence, we do not report any functional assay to validate them in this study.

The finding of these four genes recapitulating HSCR in zebrafish clearly demonstrates that genes that would have never been followed up based on the usual gene selection criteria should not be ignored. Using bioinformatics prediction and statistics, we would have focused on *RET* and *CKAP2L* as they were the only genes significantly enriched for rare variants in patients.

We wondered whether any of these four genes could be linked to the ENS or whether they play relevant roles in neuronal development or NC-derived cell types in general. In fact, by studying these genes in more depth we noticed that all four, despite a lack of obvious connection to the known ENS pathways, are involved in the development of the CNS or the NC, making these not as random as they might first appear.

DENN/MADD domain containing 3 (*DENND3*) is a guanine nucleotide exchange factor (GEF) that is involved in intracellular trafficking by activation of the small GTPase RAB12 [25]. In zebrafish, Rab12 and other Rab GTPases are highly expressed by pre-migratory NC cells and their expression is dysregulated in Ovo1 morphant zebrafish that display altered migration of NC cells [26]. Independently of *RAB12*, *DENND3* also regulates Akt activity, which is involved in the proliferation and survival of enteric NC cells [25, 27].

Nicalin (*NCLN*) is a key component of a protein complex that antagonizes Nodal signaling [28], which in vertebrates is involved in induction of the mesoderm and endoderm [29]. In contrast, inhibition of Nodal signaling is required for the specification of human embryonic stem cells into neuroectoderm, including the NC [30, 31]. The antagonizing function of Nicalin on Nodal signaling is therefore consistent with the NC specification required for ENS development.

*NUP98* encodes a precursor protein that is autoproteolytically cleaved to produce two proteins: NUP98 from the N-terminus and NUP96 from the C-terminus [32]. A missense DNM was identified in the last exon of the *NUP98* gene and therefore affects the NUP96 protein. As in humans, zebrafish Nup96 is produced by cleavage of the Nup98 precursor protein. Since morpholinos act on the mRNA level, both *nup98* and *nup96* were targeted in our zebrafish experiments. It is therefore unclear whether the observed aganglionosis is caused by loss of Nup98 or Nup96. NUP96 is one of approximately 30 proteins in the nuclear pore complex (NPC) [33] and its expression level regulates the rate of proliferation [34]. Two other members of the NPC (*Nup133* and *Nup210*) are involved in neural differentiation in mice [35, 36]. Moreover, NUP96 interacts with NUP98 and NUP98 is involved in transcriptional regulation of the HSCR genes *SEMA3A*, *DSCAM*, *NRG1*, and the *NRG1* receptor *ERBB4* in human neural progenitor cells [37]. Therefore, it is likely that loss of both NUP proteins (NUP96 or NUP98) could contribute to HSCR development.

The mouse ortholog of *TBATA* (Thymus, brain and testes associated) is called Spatial and is highly expressed during differentiation of several tissues [38]. These include the cerebellum, hippocampus, and Purkinje cells in the brain, where TBATA/Spatial is expressed in early differentiating neurons [39]. In mouse hippocampal neurons, *TBATA*/Spatial is required for neurite outgrowth and dendrite patterning [40].

**Fig. 3:**
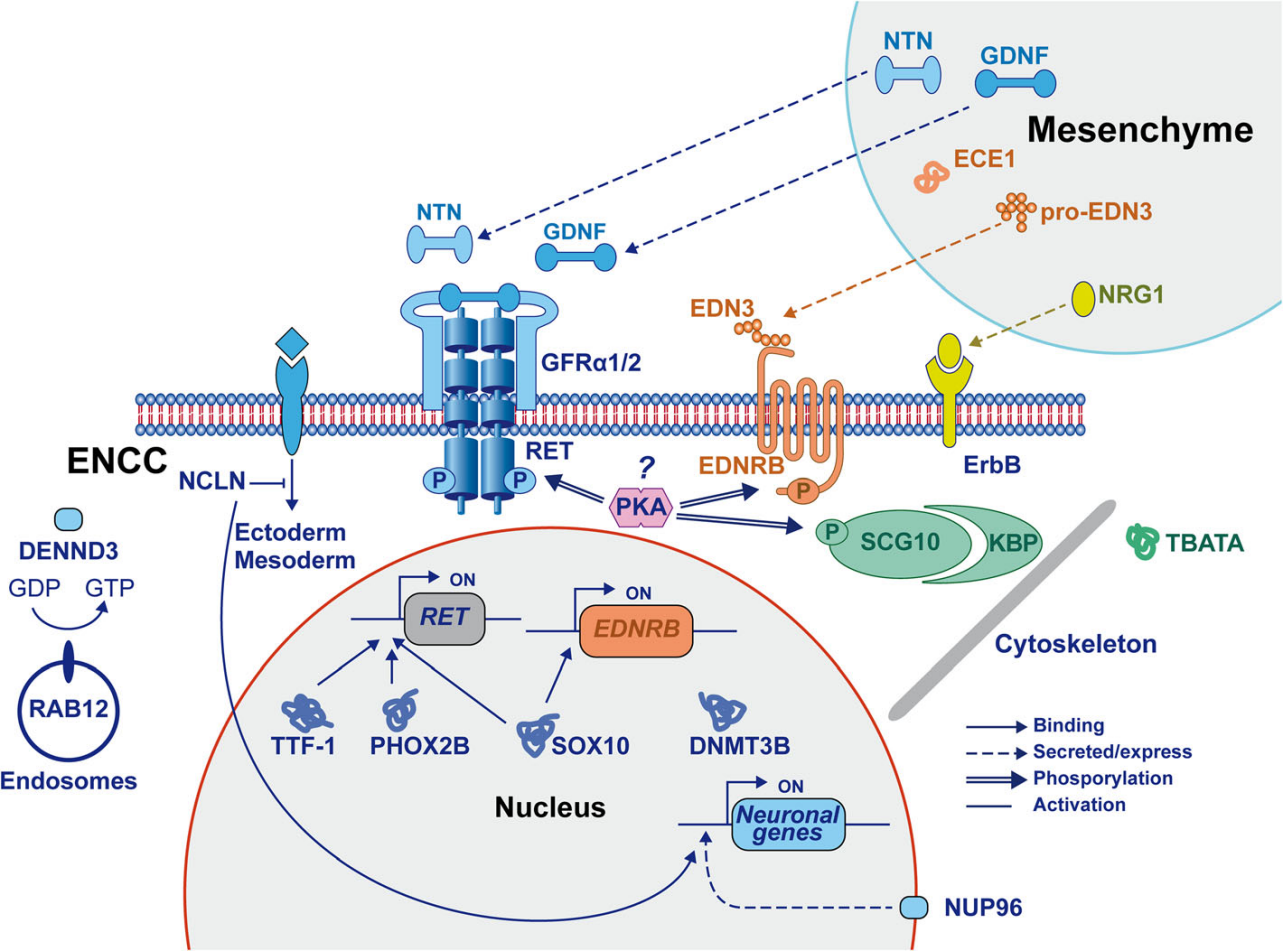
Newly identified genes in ENS development. All symbols represent proteins coded by genes known to be involved in HSCR or novel genes identified in this study. The effect of *NUP98* is shown by protein NUP96. The interaction effects between different proteins are illustrated by four different lines representing binding, secreted/express, phosphorylation, and activation. *ENCC* enteric neural crest cell

The four newly identified HSCR candidate genes seem to play a role in neuronal development and could potentially be involved in HSCR (Fig. 3). This also suggests a clear link between CNS and ENS development. This does not come as a total surprise, as several known HSCR genes (e.g., *KBP*, *SOX10*, *NRG1*, *IKBKAP*, *ZEB2*, *PHOX2B*) are involved in both CNS and ENS pathologies [2, 41–43] and the fact that HSCR is strongly associated with Down syndrome.

Besides the fact that several HSCR/neuromuscular genes are known to be associated with CNS defects, the opposite is also described. Many neurological and psychiatric disorders are associated with constipation, and sometimes defects in the ENS are reported [44]. For instance, it has recently been described that mutations in *CDH8* result in a specific subtype of autism in combination with gastrointestinal problems. A *cdh8* ^−*/*−^ zebrafish recapitulates the human phenotype, including increased head size, impairment of gastrointestinal motility, and reduction in post-mitotic enteric neurons [45]. Besides, searching “CNS” and “autism” in Phenolyzer (http://phenolyzer.wglab.org/) returns two ENS genes, *APP* [46] and *MECP2* [47]*.*

Given the fact that HSCR occurs together with neurological disorders more often than expected, it is not surprising that dysfunction of these newly identified neurological-related genes results in dysregulation of the NC-derived cells that form the ENS, and hence in HSCR. These data are further corroborated by the expression patterns we observed for the orthologs of these four genes in zebrafish embryos (Fig. 2), with all four being expressed in both brain and gut.

Using zebrafish as a model we have experimentally shown that some of the genes with de novo mutations appear to be functionally or biologically linked with the ENS. As the effect of de novo variants on the phenotype may depend on the genetic background, it is tempting to speculate that those genes that failed to reproduce the HSCR phenotype in zebrafish could, in fact, contribute to the disease in humans.

While statistical evidence of involvement in HSCR has only been attained for RET, the functional studies presented here support the possible contribution of DENND3, NCLN, NUP98, and TBATA to the disorder. Finding a role for these genes in ENS development will open new research avenues and enhance our knowledge about ENS development and HSCR disease mechanisms. Until now, we believed that the number of cellular processes involved in the development of HSCR was limited. Clearly this idea needs to be revisited as the novel genes we identified are not directly linked to any of the currently known HSCR gene networks.

## Conclusions

Our data open new fields of investigation into HSCR pathology and provide insight into the development of both the ENS and CNS. Moreover, as a lesson for all those who work on rare heterogeneous diseases, the study demonstrates that functional analyses of genes carrying DNMs is warranted to delineate the full genetic architecture of rare complex diseases.

## Methods

### Study samples

#### Trios

A total of 24 trios (affected child and unaffected parents) without family history of HSCR recruited in five different centers were included for whole exome sequencing (WES). The patients were all non-syndromic. Five trios were of Chinese origin whereas 19 were of Caucasian ancestry. We prioritized the most/more severe and rarer HSCR cases for this study, namely female patients with long segment or total colonic aganglionosis. Sixteen out of the 24 patients had previously tested negative for *RET* damaging variants by traditional technologies. Characteristics of the patients are presented in Additional file 1: Table S1. Informed consent was obtained from all participants.

#### Case–control

WES data from 28 additional sporadic HSCR patients without sub-phenotype limitation (singletons) and 212 controls were used to check gene recurrence and assess the gene burden for rare variants (Additional file 1: Table S1).

### Data generation

#### Whole exome sequencing

DNA samples were sequenced in four centers. The exome-capture kit and sequence platforms used per center are detailed in Table S2. Appropriate mapping tools (Burrows–Wheeler aligner (BWA) for Illumina data and Bfast for Solid data) were used to align sequence reads to the human reference genome (build 19) [48]. Sequence quality was re-evaluated using the FastQC toolbox, Picard’s metric summary, and the Genome Analysis Toolkit (GATK) Depth-of-Coverage module. After initial quality control (QC) all eligible sequences were pre-processed for local indel realignment, PCR duplicate removal, and base quality recalibration [49].

#### Genome-wide SNP array

To determine copy number variants (CNVs) and regions of homozygosity, DNA was hybridized to the HumanCyto SNP12 BeadChip (Illumina, San Diego, CA, USA) according to standard protocols.

### Variant calling and prioritization

Aligned reads from all sequenced samples were pre-processed according to standard guidelines [49]. Variant calling was done independently for Illumina reads or Solid reads using the GATK unified Genotyper 2.0 [50]. To avoid mismatched regions across different capture kits, calling was performed whole genome-wide without limiting to any capture array. A special setting (allow potentially miscoded quality scores) was used to make color-spaced solid reads compatible to the program (Broad institute). Raw variants (including single nucleotide variants and short insertions/deletions) with individual genotypes and their affiliated quality scores were stored in a standard VCF format after calling. Quality assessment (QA) and QC were then adopted on a few sets of variants (raw variants, exonic variants, rare variants) to generate a confident variant set for downstream prioritization (Additional file 1: Supplementary methods). A clean variant set at exonic regions was produced after variant-level and genotype-level QC. Rare coding sequence variants were then prioritized by filtering out those variants with minor allele frequency >0.01 in any of these public databases (dbSNP137, 1000 Human Genome project, and NHLBI Exome Sequencing project). An automatic pipeline integrating GATK [49], KGGSeq [51], Annovar [52], and Plink [53] was used to generate the final set of qualified variants (Additional file 2: Figure S1).

### Identification of DNMs

#### WES DNM detection

Rare, exonic variants present in the probands but absent in both parents were considered DNMs. To select putative DNMs (or de novo variations) the following criteria were used: 1) minimal coverage of five in patients and parents; 2) a minimal genotype quality score of 10 for both patients and parents; 3) at least 10% of the reads showed the alternative allele in patients; and 4) not more than 10% of the reads showed the alternative allele in parents. Subsequently all remaining DNM variants were manually inspected using the Integrated Genome Viewer (IGV) and classified into five different confidence ranks according to their base-calling quality and strand bias. The first two ranks of DNM candidates were selected for validation by Sanger sequencing; while the other three classes of candidates were re-evaluated by a model trained from variants submitted for Sanger sequencing (Additional file 2: Supplementary methods).

#### RET gene inspection

To guarantee that no de novo mutations had been missed in the major HSCR gene, the depth of coverage of each of the 21 exons of *RET* was manually inspected for each patient. All exons with a coverage <10 were Sanger sequenced. Mutation Detector software (Thermo Fisher Scientific) was used to identify rare coding sequencing mutations from raw Sanger sequences; any mutation found in a trio proband was further checked in his/her parents. Besides rare mutations, bi-allelic genotypes for the common risk single nucleotide polymorphisms (IVS1 + 9494, rs2435357T) were extracted from local databases or newly genotyped.

#### CNV detection

The Nexus®software program (Biodiscovery, El Segundo, CA, USA) was used to normalize and analyze the SNP array data as mentioned above. Loss is defined as the loss of a minimum of five probes in a 150-kb region, with a minimum log R ratio of –0.2. Gain is defined as the gain of a minimum of seven probes in 200-kb region, with a minimum log R ratio of 0.15. The minimum length of regions of homozygosity analysed was 2 Mb. The identified CNVs were reviewed for pathogenicity using the UCSC genome browser (http://genome.ucsc.edu/), the DGV database (http://dgv.tcag.ca/dgv/app/home), the Decipher database (https://decipher.sanger.ac.uk/), and our in-house local reference database that consists of 250 healthy controls and 250 individuals of the general population.

### Statistical tests

#### De novo *mutation rate*

All proven DNMs were classified into loss-of-function (nonsense single nucleotide variants (SNVs), frame-shift indels and splicing sites), missense SNVs, in-frame indels, and synonymous SNVs. The counts of DNM per trio were fitted to a Poisson distribution with lamda as observed mean. De novo mutation rates were calculated for these DNM subtypes and compared to 677 published healthy trios and neurodevelopmental disease trios using a binomial test [6–8, 10, 11, 54]. Given the per-gene mutation rate in Samocha et al. [13], statistical over-representation of mutations in all 24 genes were calculated using Fisher’s exact test.

#### Gene-wide burden analysis

Genes with DNMs were further scrutinized for the presence of inherited rare damaging variants in the trios as well as in HSCR singletons for whom WES data were available. A detailed analytical protocol was shared before running the association in each center. Briefly, genotypes of rare damaging variants (as previously defined) in genes carrying one or more de novo mutations were extracted from raw sequencing reads. The CMC test in the Rvtest package was used to collapse multiple variants into the same gene (boundary defined using hg19 refgene) and compare overall burden between cases and local matched controls [55]. *P* values were estimated by asymptotic chi-square distribution. The gene-wise *p* value, burden direction, and variant count per gene were exported. Ultimately, the sample size weighted Z-score method was used to conduct meta-analysis on gene-wise summary statistics from three centers using the same protocol.

### Bioinformatics analysis

#### Variant-level implication

The impact of each DNM to its carrying gene was predicted using several bioinformatics tools or databases. The conservation of missense SNVs was predicted using GERP and PhyloP across 29 different species. The deleteriousness of missense or nonsense SNVs were determined by a logit model incorporating five prediction programs (Polyphen2, Sift, MutationTaster, PhyloP, and likelihood ratio) [24]. Human Splicing finder was used to predict whether DNMs causing synonymous change or locating at splicing sites (exon ±2 bp) created or disrupted splice sites [56]. To assess if synonymous DNMs had any effect on the transcripts, we used RNAmute [57]. Finally, ClinVar and PubMed were searched for the same or similar mutations in the same gene that present in healthy controls or other disease patients.

#### Gene-level implication

Evidence of gene-level implication was collected from two aspects. First, those 24 genes carrying DNMs were searched against databases (the ATGU’s server (https://github.com/michaelchess/gene-lookup) and ExAC browser (http://exac.broadinstitute.org/)) for recurrence and constrained scores [13, 17]. Second, ENS candidate genes/gene sets were curated (Additional file 1: Supplementary methods) [58–60] and then linked to newly identified genes using pathway or protein–protein interaction network information. Disease Association Protein–Protein Link Evaluator (DAPPLE) was used to test whether the genes carrying DNMs in our study are functionally connected to each other. The significance of observed pathway enrichment and network connectivity was evaluated empirically using randomly selected genes from among genes of the same genomic size as the identified DNM genes. InWeb and Ingenuity Pathway Analysis were used to detect direct and indirect protein interactions between ENS-related genes and genes with DNMs.

### Gene expression in the ENS

In order to test the involvement of the newly identified genes in ENS development, in-house expression data were shared from other parallel projects in the Hong Kong and Rotterdam centers. The first expression dataset was from RNA sequencing on an iPSC-induced enteric neural crest cell (ENCC) for a HSCR patient; the second and third expression datasets were from microarray chips on embryonic mouse gut and ENCC.

### Zebrafish

*Tg(-8.3phox2b:Kaede)* transgenic zebrafish (*Danio rerio*) embryos were obtained from natural spawning. Maintenance of zebrafish and culture of embryos were carried out as described previously [61]. Embryos were staged by hours (hpf) or days (dpf) post-fertilization at 28.5 °C.

#### Gene knockdown by antisense morpholinos

Antisense morpholinos (MOs; Gene Tools LLC) targeting the zebrafish orthologs of the candidate genes, by blocking either translation or splicing, were microinjected to one- to four-cell stage *Tg(-8.3phox2b:Kaede)* transgenic zebrafish embryos as previously described [19]. For candidate genes that are duplicated in the zebrafish genome, morpholinos targeting all paralogs were co-injected. A standard control MO and 5-nucleotide mismatch control MOs for *ckap2l*, *dennd3a*, *dennd3b*, *ncl1*, *nup98*, and *tbata* were used as negative control. Embryos were raised to 5 dpf, analyzed, and imaged under a stereo fluorescence microscope (Leica MZ16FA and DFC300FX). An HSCR-like phenotype was defined as the absence of enteric neurons in the distal intestine in 5-dpf embryos. Sequences and dosages of all MOs used are included in Additional file 10: Table S9.

#### Gene knockout by CRISPR/Cas9

The design and synthesis of gRNA were carried out as described [62]. Briefly, CRISPRscan (http://www.crisprscan.org/) was used to design gRNA sequences against *ckap2l*, *dennd3a*, *dennd3b*, *ncl1*, *nup98*, and *tbata*. Designs with high predicted efficiency and low predicted off-target effects were chosen. DNA templates for gRNA synthesis were generated by PCR fill-up reaction [62] and the gRNAs were synthesized using a MEGAshortscript™ T7 Transcription Kit (Invitrogen). gRNA (150 pg) was co-injected with recombinant 667 pg Cas9 protein to one-cell stage *Tg(phox2b:kaede)* embryos. For *dennd3a* and *dennd3b* knockout 75 pg of each gRNA was co-injected with Cas9. The injected larvae and un-injected control were cultured to 5 dpf for phenotype checking and imaging. Each larva was collected separately for genomic DNA extraction and T7E1 assay to confirm the presence of indel mutation as described previously [21]. In brief, the genomic region flanking the gRNA target site was amplified by PCR. The PCR product was denatured and slowly re-annealed to allow the formation of a heteroduplex. The re-annealed PCR product was digested with T7 endonuclease I (New England Biolab) at 37 °C for 45 min and then resolved by 2% agarose gel electrophoresis. The sequences of gRNAs and primers are listed in Additional file 11: Table S10.

#### Expression analysis

To confirm the target genes were successfully knocked down, total RNA was extracted from 1-dpf embryos (*n* = 50) injected with the splice blocking MO using RNA Bee (Amsbio) and cDNA were reverse transcribed using a iScript cDNA Synthesis Kit (Bio-rad). qPCRs were performed using a KAPA Sybr® Fast qPCR Kit (KAPA Biosystems; see Additional file 12: Table S11 for primer details) and the expression of the target gene was normalized by the mean expression of two housekeeping genes (*elfa* and *actb*). The relative expression of the target gene in the splice blocking MO-injected embryos to the control MO-injected embryos was determined by the Livak method [63].

To determine the temporal expression of the zebrafish ortholog, RT-PCR was performed at various time points with primers used to amplify a segment of the open reading frame of each gene. To determine the spatial expression patterns of *dennd3a*, *dennd3b*, *ncl1*, *nup98*, and *tbata*, antisense digoxigenin-labeled probes for both genes were generated and whole-mount in situ hybridization was performed as described by Thisse et al. [64].

## Additional files

Additional file 1: Table S1. Information on samples included in the study. (XLSX 10 kb)
Additional file 2: Supplementary methods and Figures S1**–**S11. (PDF 4258 kb)
Additional file 3: Table S2. Quality metrics for sequencing reads and variants from different cohorts. (XLSX 9 kb)
Additional file 4: Table S3. Statistical models for mutation prediction. (XLSX 9 kb)
Additional file 5: Table S4. Comparison of de novo mutation rates. (XLSX 9 kb)
Additional file 6: Table S5. Joint distribution of common and rare variants for each trio proband. (XLSX 13 kb)
Additional file 7: Table S6. Gene recurrence and burden test. (XLSX 14 kb)
Additional file 8: Table S7. Bioinformatics prediction of the functional impact of DNMs. (XLSX 11 kb)
Additional file 9: Table S8. Characteristics of 116 ENS-related HSCR candidate genes. (XLSX 32 kb)
Additional file 10: Table S9. Sequences and dosages of antisense morpholinos. (XLSX 10 kb)
Additional file 11: Table S10. gRNA and primers for zebrafish knockout and T7E1 assay. (XLSX 9 kb)
Additional file 12: Table S11. Primers for expression analysis in zebrafish. (XLSX 9 kb)
