# Supplementary material for: Whole exome sequencing coupled with unbiased functional analysis reveals new Hirschsprung disease genes
Source: Genome Biol. 2017 Mar 8;18:48. doi: 10.1186/s13059-017-1174-6 (PMC5343413; doi:10.1186/s13059-017-1174-6)
Supplement: Additional file 2: — Supplementary methods and Figures S1–S11. (PDF 4258 kb) [file 13059_2017_1174_MOESM2_ESM.pdf]

Supplementary Material to

Whole exome sequencing coupled with unbiased functional analysis reveals new Hirschsprung disease genes

Hongsheng Gui[1,2*], Duco Schriemer[3*], William W. Cheng[1,4*], Rajendra K. Chauhan[4], Guillermo Antiňolo[5,6], Courtney Berrios[7], Marta Bleda[6,8], Alice S. Brooks[4], Rutger W.W. Brouwer[9], Alan J. Burns[4,10], Stacey S. Cherny[2], Joaquin Dopazo[5,6], Bart J.L. Eggen[3], Paola Griseri[11], Binta Jalloh[12], Thuy-Linh Le[13,14], Vincent C.H. Lui[1], Berta Luzón-Toro[5,6], Ivana Matera[11], Elly S.W. Ngan[1], Anna Pelet[13,14], Macarena Ruiz-Ferrer[5,6], Pak C. Sham[2], Iain T. Shepherd[12], Man-Ting So[1], Yunia Sribudiani[4,15], Clara S.M. Tang[1], Mirjam C.G.N. van den Hout[9], Herma C. van der Linde[4], Tjakko J. van Ham[4], Wilfred F.J. van IJcken[9], Joke B.G.M. Verheij[16], Jeanne Amiel[13,14], Salud Borrego[5,6], Isabella Ceccherini[11], Aravinda Chakravarti[7], Stanislas Lyonnet[13,14], Paul K.H. Tam[1], Maria-Mercè Garcia-Barceló[1#] and Robert M.W. Hofstra[4,10#]

[1]Department of Surgery, Li Ka Shing Faculty of Medicine, The University of Hong Kong, Hong Kong, SAR, China

[2]Centre for Genomic Sciences, Li Ka Shing Faculty of Medicine, The University of Hong Kong, Hong Kong, SAR, China

[3]Department of Neuroscience, section Medical Physiology, University of Groningen, University Medical Center Groningen, Groningen, The Netherlands

[4]Department of Clinical Genetics, Erasmus Medical Center, Rotterdam, The Netherlands

[5]Department of Genetics, Reproduction and Fetal Medicine, Institute of Biomedicine of Seville (IBIS), University Hospital Virgen del Rocío/CSIC/University of Seville, Seville, Spain

[6]Centre for Biomedical Network Research on Rare Diseases (CIBERER), Seville, Spain

[7]McKusick-Nathans Institute of Genetic Medicine, Johns Hopkins University School of Medicine, Baltimore, USA

[8]Department of Medicine, School of Clinical Medicine, University of Cambridge, Addenbrooke's Hospital, Cambridge, United Kingdom

[9]Erasmus Center for Biomics, Erasmus Medical Center, Rotterdam, The Netherlands.

[10]Stem Cells and Regenerative Medicine, Birth Defects Research Centre, UCL Institute of Child Health, London, UK

[11]UOC Genetica Medica, Istituto Gaslini, Genova, Italy

[12]Department of Biology, Emory University, Atlanta, USA

[13]Laboratory of embryology and genetics of human malformations, INSERM UMR 1163, Institut Imagine, Paris, France.

[14]Department of Genetics, Paris Descartes-Sorbonne Paris Cité University, Hôpital Necker-Enfants Malades (APHP), Paris, France

[15]Department of Biochemistry and Molecular Biology, Faculty of Medicine, Universitas Padjadjaran, Bandung, Indonesia

[16]Department of Genetics, University of Groningen, University Medical Center Groningen, Groningen, The Netherlands

*Equally contributing authors; [#]equally contributing corresponding authors.

Corresponding author: Robert Hofstra, Department of Clinical Genetics, Erasmus University Medical Center, PO BOX 2040, 3000CA Rotterdam, The Netherlands. Tel: +31-10-7037643. E-mail: r.hofstra@erasmusmc.nl; Mercè_Garcia-Barceló, Department of Surgery, The University of Hong Kong, Hong Kong, SAR, China. Tel +852-28315073; E-mail: mmgarcia@hku.hk

SUPPLEMENTARY METHODS

Generation of ENS candidate genes

Candidate genes were selected by a literature review on Hirschsprung disease research, which included both genetic and functional studies. Most of them were also covered in Jiang et al. [58] and Gui et al. [59], which previously summarized possible genes related to HSCR or involved in ENS development. The genes were categorized into 4 major types, genes selected based on: genetic linkage, genetic association, microarray expression, and animal models. In total 116 genes were selected that fit more than 1 category (Additional file 7: Table S6). A few of these genes fall into the same pathways previously implicated in neural crest cell migration, proliferation and differentiation. Three pathways (*RET* signaling pathway, *EDNRB* signaling pathway and *KBP* signaling pathway) were key partners involved in ENS development [60].

Quality assessment and control for exome variants

Concrete criterions in quality assessment (QA) include: total number of variants; dbSNP137 coverage; Transition/Transversion (Ti/Tv) ratio; genotype concordance rate and cross-sample identical-by-decent (IBD) relatedness [53]. Two complementary steps were applied in quality control (QC), including variant-level filtering (hard filtration or variant quality recalibration (VQSR)) and genotype-level filtering. In detail, we annotated GATK-called variants as low quality SNPs (“QD <2.0” or "MQ <40.0" or "FS >60.0" or "HaplotypeScore >13.0" or "MQRankSum <-12.5" or "ReadPosRankSum <-8.0" in their ‘info’ field) and low quality Indels (“QD <2.0" or "ReadPosRankSum <-20.0" or "InbreedingCoeff <-0.8" or "FS >200.0 in ‘info’ field); in addition, VQSR differentiated a few relatively low quality SNVs (labeled as “TruthSensitivityTranche99.90to100.00” after Gaussian mixture modeling at true sensitivity 99%) from other passed SNVs. On the other hand, individual genotypes were evaluated by quality

parameters in the field of genotyping, mainly reflecting the likelihood of three possible genotypes (reference homozygous, heterozygous and alternative homozygous). A heterozygous genotype was kept only if it was supported by >4 total reads, and the ratio for alternative allele is above 0.25. Comparatively, a reference or alternative homozygous genotype was accepted if it was supported by > 4 total reads, and ratio for reference or alternative allele is above 0.95.

Mutation validation and prediction

Each DNM candidate was manually inspected using the Integrative Genomic Viewer (IGV) and they were categorized into five different groups: probably true positive, possibly true positive, unclear, possibly false positive and probably false positive. Two lists of putative DNM candidates were generated for confirmation by Sanger sequencing. The first list contains 74 variants with high confidence ranking (probably true positive and possibly true positive). Raw data were then re-evaluated to generate 48 candidates with relatively low-confidence (unclear), especially for those trios without any confirmed DNM in the first round. Rare (minor allele frequency < 0.01 in public databases) predicted damaging variants in genes carrying confirmed *de novo* mutations were extracted from exome calls and submitted for Sanger validation. The allele origin was determined by checking the mutation site in both parents. Phasing of DNM and inherited variants in the same gene was also performed by Sanger sequencing. Rare damaging inherited variants located in 116 ENS candidate genes were extracted from exome reads using the same pipeline (Additional file 2: Figure S2); and the transmission patterns of these variants were determined by referring to parental and maternal genotypes at the same site.

Stepwise logistic regression was used to select effective predictors of the *de novo* status in a trio and for the presence or absence of a mutation in a given individual. The performance

of these prediction models was evaluated using 10-fold cross validation by the software WEKA. For model fitting to DNM status in the trios, genotype quality (represented by normalized phred likelihood score for the second most likely genotype) in the child and alternative allelic ratio in the parents were prioritized. The **A**rea **U**nder the Receiver Operating Characteristic **C**urve (**AUC**) was 0.959 (Additional file 4: Table S3) which suggests that the model predicts the DNM status accurately. This model was then adopted to test all other unvalidated *de novo* candidates (falling under the ”unclear”, “possibly false positive” or “probably false positive” categories), which all turned out to be negatives. For model fitting to the presence or absence of a variant in the patients, genotype quality and alternative allelic ratio in each individual were retained. The AUC was 0.824 (Additional file 4: Table S3). This second model was then used to help predict the presence of rare variants in the DNM genes or ENS genes. Only those variants predicted as positive candidates were shown (Additional file 6: Table S5).

SUPPLEMENTARY FIGURES

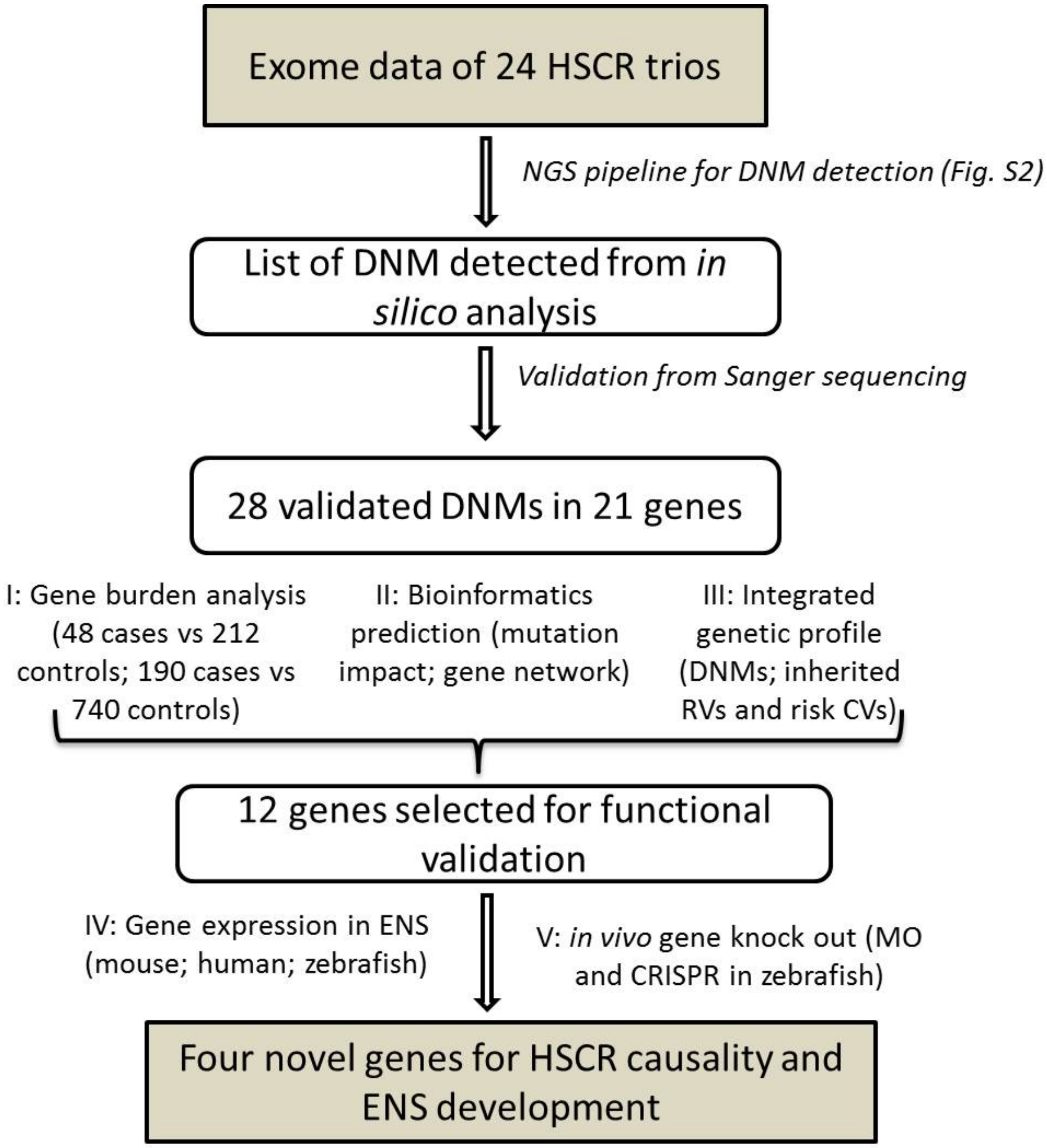

Figure S1 Flow chart of the study design

I: statistical evidence from gene-wise burden analysis (detail in Additional file 7: Table S6); II: bioinformatics prediction of the mutation impact and the gene network (detail in Additional file 8: Table S7 and Additional file 2: Figure S7); III: mutation profile (*de novo* mutations, rare damaging variants in ENS candidate genes, and risk *RET* enhancer common SNP) for each patient (detail in Additional file 6: Table S5); IV: gene expression analyses (detail in Table 2, Figure 2 and Additional file 2: Figure S7-S9); V: in vivo zebrafish analyses (Figure 1, Additional file 2: Figure S10).

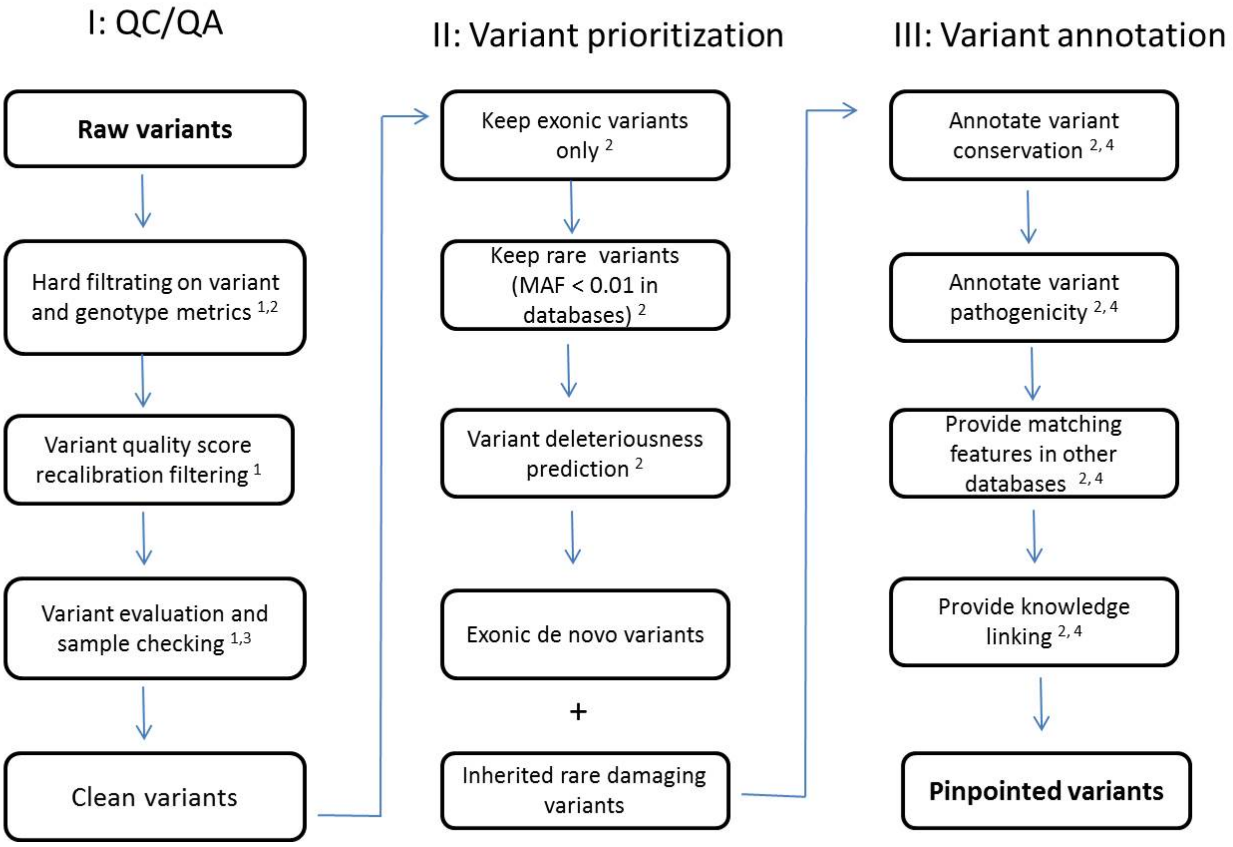

Figure S2: Analytical pipeline for exome sequence filtration and prioritization

[1] GATK: GATK is used for variant hard filtering, variant quality score recalibration and variant evaluation.

[2] KGGSeq: KGGSeq is used for variant filtering, deleteriousness prediction and variant/gene annotation by additional knowledge (STRING, MsigDB, and PubMed).

[3] PLINK: PLINK IBS/IBD sharing is used to estimate the sample relationship.

[4] ANNOVAR: Annovar is mainly used to double-check the final remaining variant for annotation, and provides supplementary features from Database of genomic variation (DGV) and clinical variation database (ClinVAR).

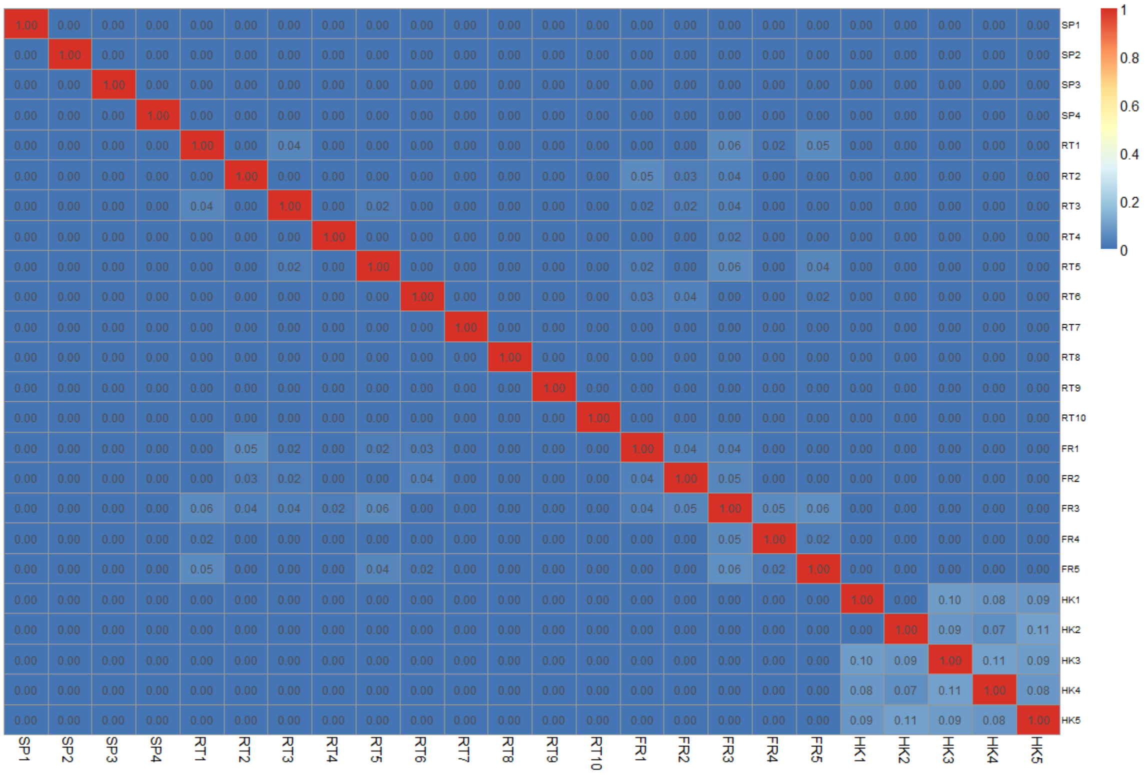

Figure S3: Relatedness plotting of HSCR exome sequences

Around 17K common SNPs (minor allele frequency > 0.01 in 1000Genomes European populations) were used to calculate identical by descent (IBD) and identical by state (IBS) proportion. Each cell shows pi_hat statistics [53] (IBD proportion, calculated from P(IBD=2)+0.5*P(IBD=1); http://pngu.mgh.harvard.edu/~purcell/plink/ibdibs.shtml) between two patients. No pairwise pi_hat coefficients are above 0.125 (the first cousin relationship); the light blue cells represent 0.07~0.11 for samples mainly from HK population, which is expected to be different from other European patients.

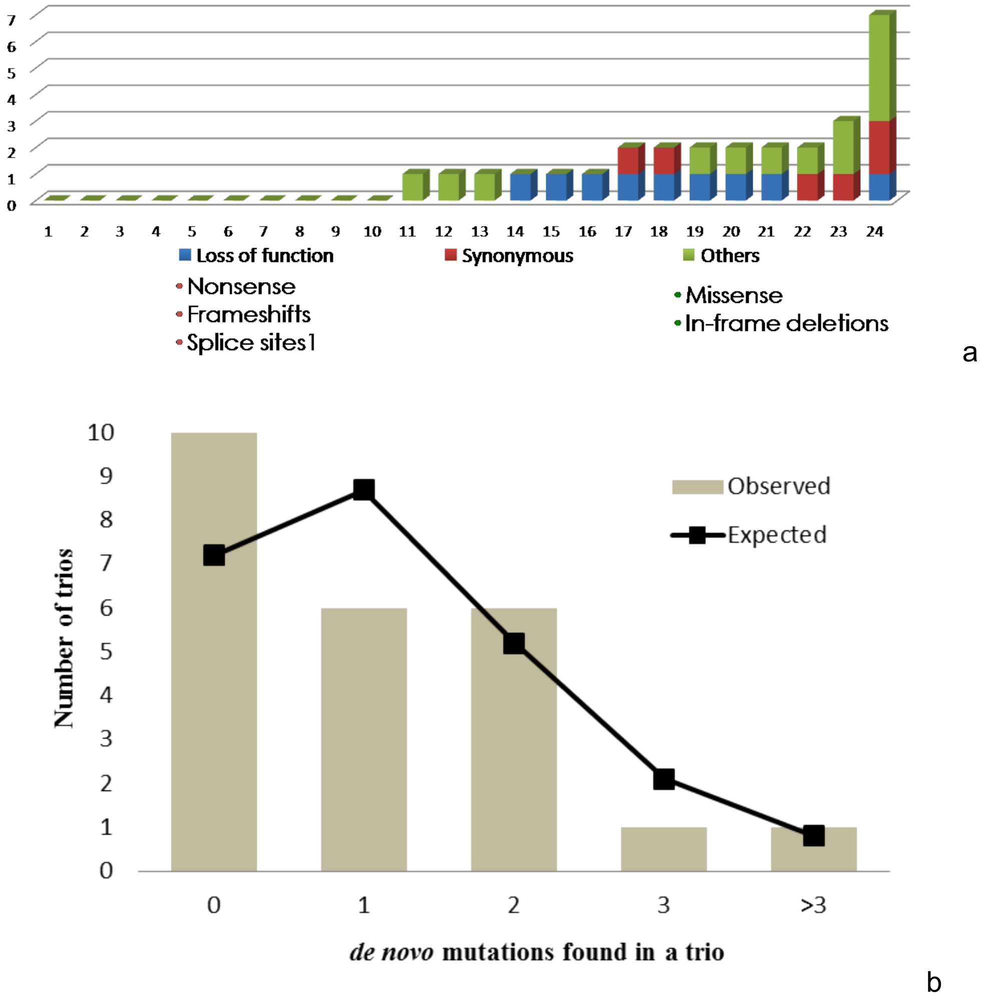

Figure S4: Distribution of *de novo* mutations per trio

(a) Number of DNMs (separated by mutation type) in each trio, categorized into three different types (Loss of function, synonymous and others); (b) Distribution of observed counts of DNMs per trio and expected counts per trio calculated from Poisson distribution (lambda at 1.2)

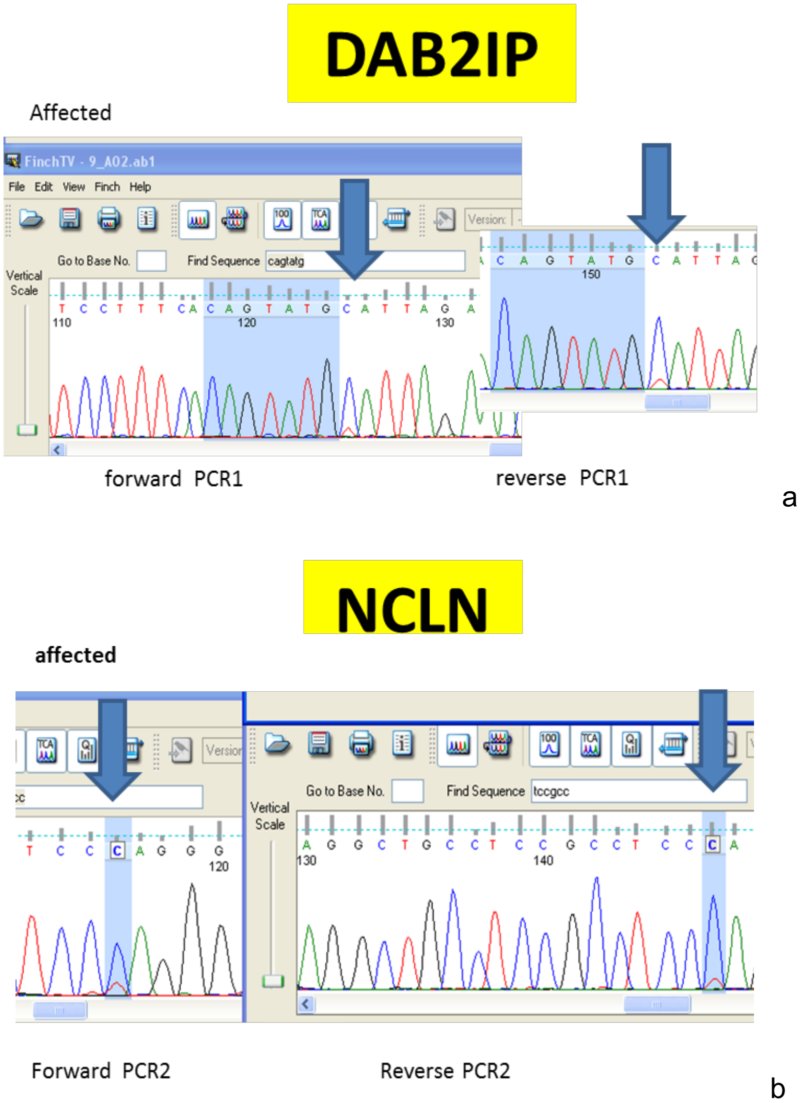

Figure S5: Sanger confirmation of mosaic DNMs for *NCLN* and *DAB2IP*

Two out of 28 *de novo* mutations (in *NCLN* and *DAB2IP* respectively) were confirmed as mosaic mutations by Sanger sequencing (forward and reverse). (a) Peak for the *DAB2IP* heterozygous mosaic mutation, (b) peak for the *NCLN* heterozygous mosaic mutation.

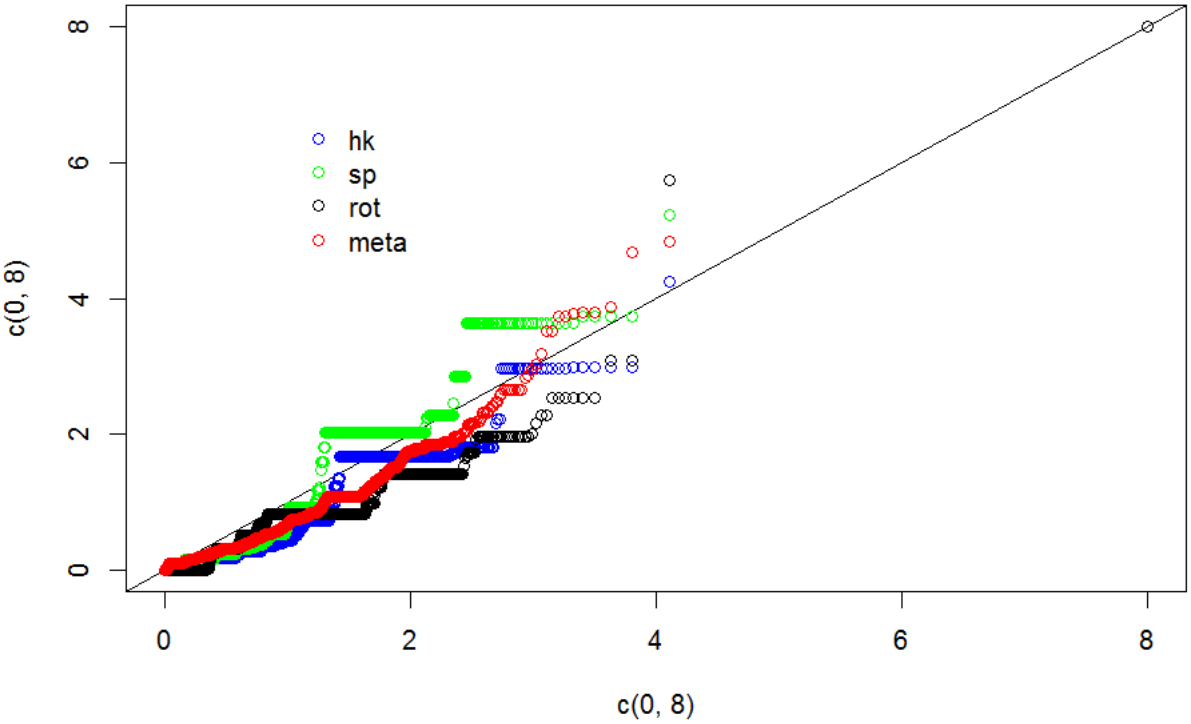

Figure S6 QQ-plot for p-values from gene burden tests

Genomic inflation coefficients for 4 different lines: hk (Hong Kong centre) 0.8419, sp (Spain centre) 0.7392, rot (Rotterdam centre) 0.2177, meta (overall meta-analysis) 0.8847.

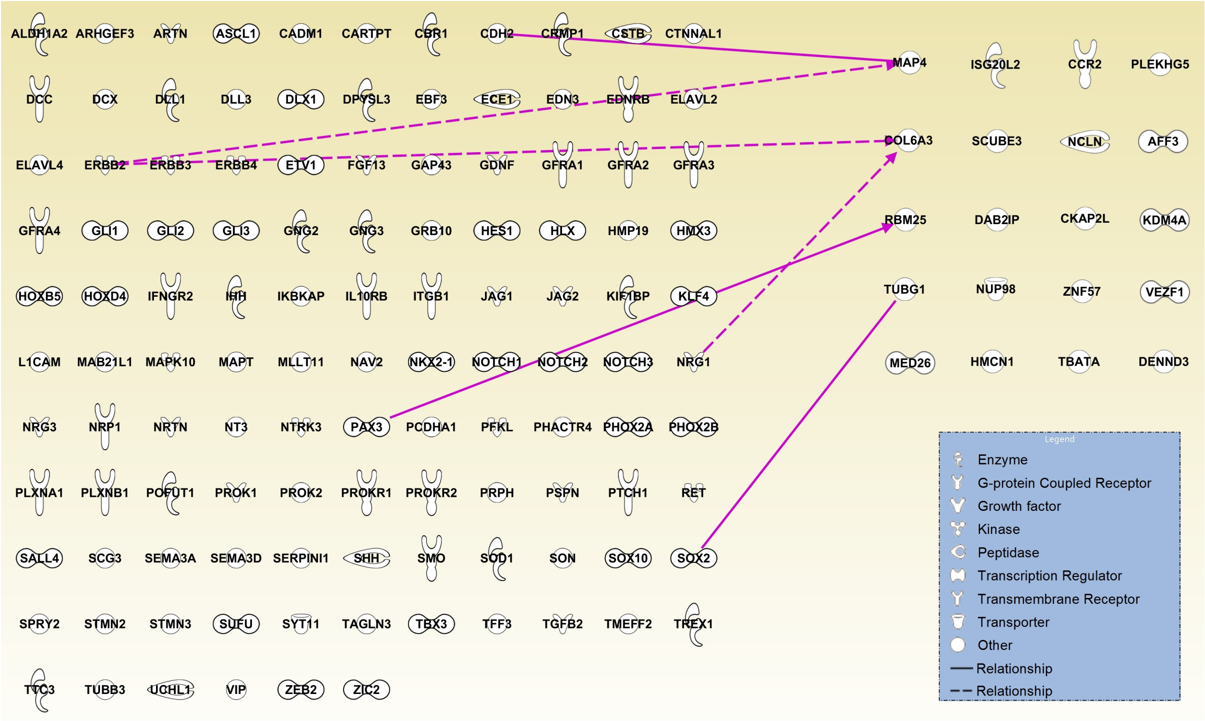

Figure S7: Connection of DNM genes and ENS genes at pathway/network level

Ingenuity Pathway Analysis (IPA) was used to link 116 ENS candidate genes (left, Additional file 9: Table S8) with the 20 newly found genes harboring *de novo* mutations (right). Solid and dotted lines represent direct and indirect interactions, respectively.

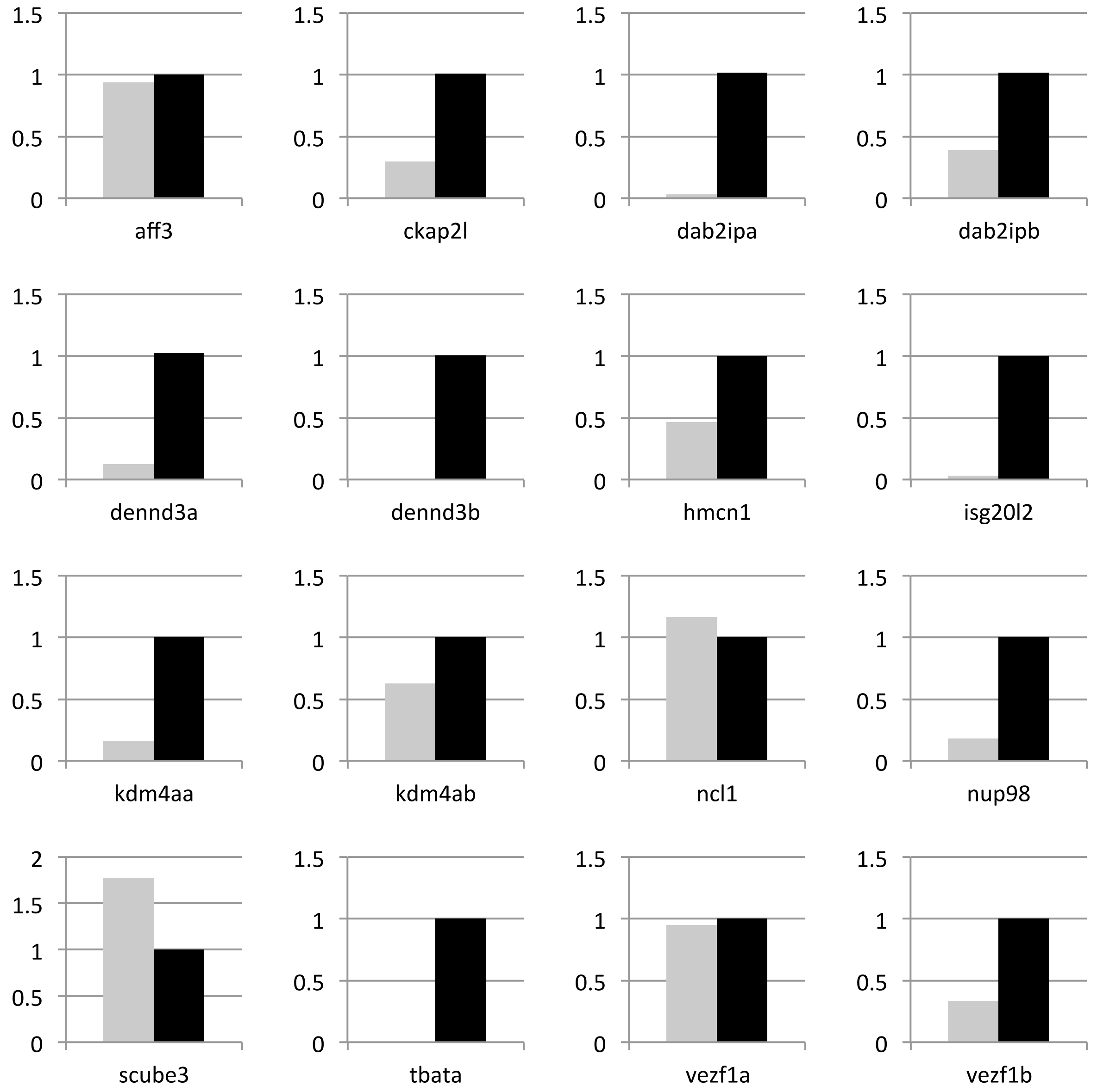

Figure S8: qPCR confirmation of gene knockdown by SBMO

Relative expression of the candidate genes between SBMO-injected (black bar) and control morpholino-injected embryos (grey bar) by qPCR.

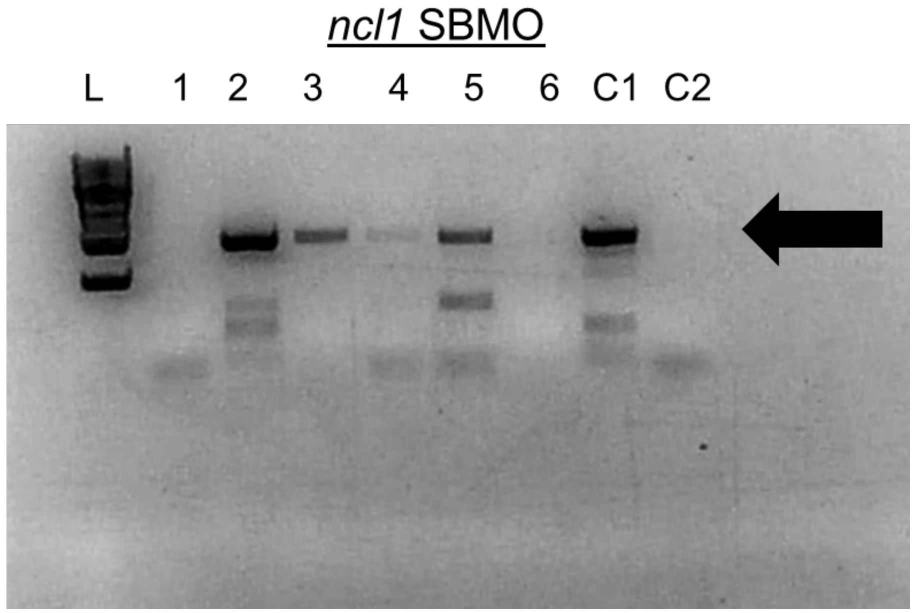

Figure S9: RT-PCR confirmation of *ncl1* SBMO knockdown

ncl1 expressions in six 1dpf embryos injected with *ncl1* SBMO were compared to control MO injected embryos. Arrow indicated the expected amplicon. L: ladder; C1 control MO injected embryo; C2: RT negative control.

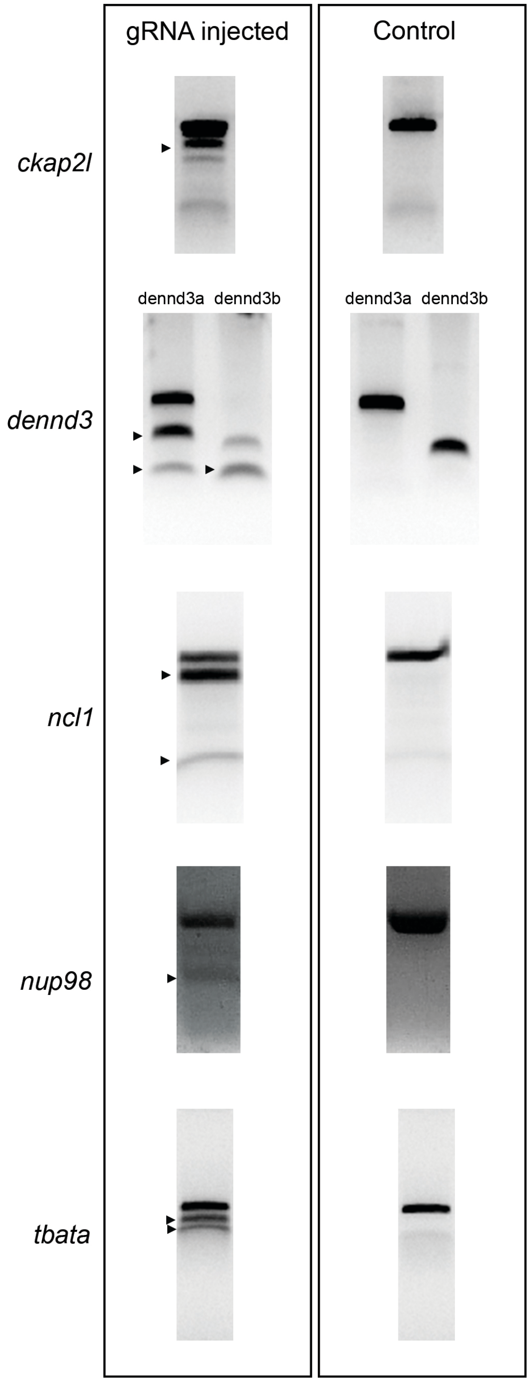

Figure S10: T7E1 detection of indel mutation in gRNA injected zebrafish
Representative gel images of T7E1 assay for the detection of indel mutation in larvae injected with CRISPR gRNA. Arrowheads indicated the extra bands resulted from T7 endonuclease I digestion of heteroduplex when indel mutation were present at the target sequence.

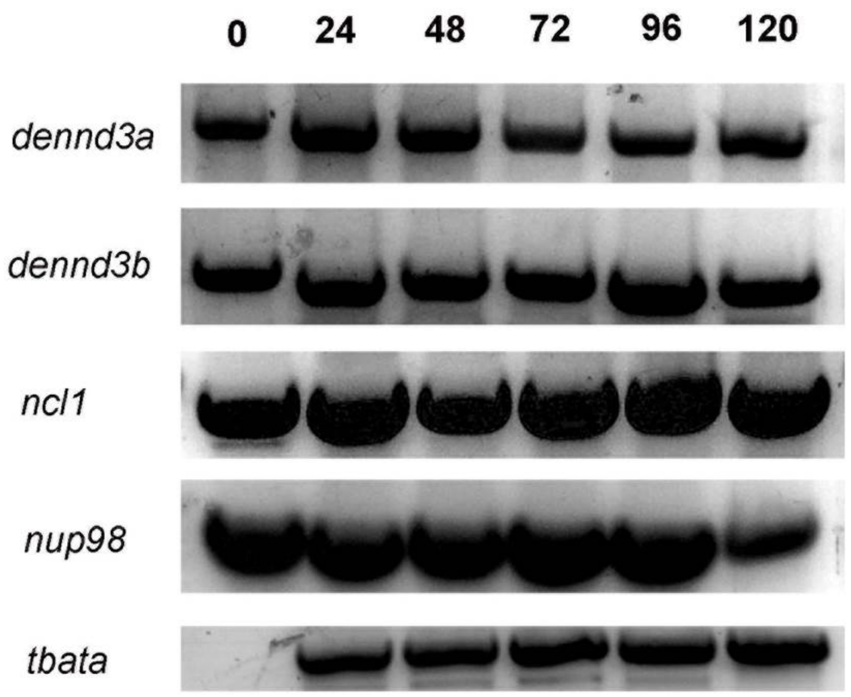

Figure S11: RT-PCR for expression of 4 candidate genes in zebrafish

Temporal expression pattern of zebrafish orthologue genes. RT-PCR for *dennd3a, dennd3b, ncl1, nup98 and tbata* was undertaken using RNA isolated from wild type embryos at 0, 24, 48, 72, 96 and 120 hpf.
